# Care home quality and ‘inappropriate’ emergency healthcare use—failing to engage with complexity

**DOI:** 10.1093/ageing/afaf030

**Published:** 2025-02-24

**Authors:** Fawn Harrad-Hyde, Jennifer Kirsty Burton

**Affiliations:** Population Health Sciences, University of Leicester, George Davies Centre, University Road, Leicester, LE1 7RH, UK; LOROS Hospice Centre for Excellence, Groby Road, Leicester, LE3 9QE, UK; Academic Geriatric Medicine, School of Cardiovascular & Metabolic Health, College of Medical, Veterinary & Life Sciences, University of Glasgow, Glasgow Royal Infirmary, Room 2.42, New Lister Building, 10 Alexandra Parade, Glasgow, G31 2ER, UK

**Keywords:** Transfer, admission, avoidable, inappropriate, care home, older people

## Abstract

Care home residents are more likely to experience unplanned, emergency healthcare than those living elsewhere in the community. This can be potentially burdensome for both individual residents and healthcare systems. Academic and policy attention has focussed on quantifying this resource use, exploring potential causes and reducing unplanned emergency healthcare. Considerable attention has been placed on emergency healthcare that may be ‘inappropriate’ or ‘avoidable’. In this commentary, we problematise such concepts. We suggest that the absence of an agreed definition can lead to wide variation in estimations which fail to acknowledge the complexity of care home residents’ needs and their right to timely healthcare support. We draw attention to the impact of popular discourse in this area as potentially stigmatising to care home organisations, staff and residents. We argue that emergency healthcare use data alone is insufficient to measure and evaluate the quality of care provided by care homes. We propose a move away from over-simplistic distinctions, between emergency healthcare that is or is not ‘appropriate’, to develop more nuanced conceptualisations that account for the complexity involved in providing healthcare support to people living in care homes. People living in care homes have significant healthcare needs. It is necessary that models of care supporting homes are resourced to respond and support care home professionals. Rather than focusing solely on quantifying emergency healthcare use, acknowledging and embracing complexity will help to refocus our efforts and redesign our services around both individual and population needs, rather than around existing systems.

## Key Points

- It is often assumed that quality care in a care home can prevent ‘inappropriate’ emergency healthcare use.
- However, an agreed definition of ‘inappropriate’ emergency healthcare is lacking and therefore estimates of this phenomenon vary.
- Care home residents depend on the wider healthcare system to respond to health deteriorations.
- Over-simplistic language neglects the complexity involved in decision-making and is stigmatising to care home residents and people who seek emergency care for them.
- We advocate prioritising routine collection of person-centred outcome and experience measures, alongside existing data.

## Introduction

International increases in unplanned, emergency hospital attendances and admissions, discussed together here as ‘emergency healthcare use’, have been described as both a patient safety problem and an international public health concern [1]. Set within the context of concerns about whether healthcare systems can support ageing populations, the emergency healthcare use of care home residents is of interest worldwide. Older people are more likely to seek emergency healthcare than younger people and care home residents are more likely to experience emergency healthcare than those living elsewhere in the community [2]. Effort has been made to quantify, explore potential causes of and reduce emergency healthcare use amongst care home residents, particularly instances considered ‘inappropriate’ or ‘avoidable’ [2–4].

It is proposed that high quality social care should prevent crises that lead to unplanned, emergency healthcare use [2]. However, care home residents live within a wider system and therefore, emergency healthcare use may reflect quality or access issues elsewhere in the healthcare system. Whilst higher-than-expected emergency healthcare use could highlight concerns about the quality of care within a care home, so too can lower-than-expected emergency healthcare use, if residents cannot access the healthcare they require [2]. Comparisons between homes rarely account for differences in resident characteristics, such as age, long-term conditions, dependency and complexity of care needs. Despite this, popular discourses and quality improvement initiatives focus on *reducing* emergency healthcare use from care homes. Much of this well-intentioned work acknowledges both resource implications and the potential for residents to be harmed during instances of emergency healthcare. Such harms can include a decline in physical or cognitive function and increased risk of mortality, which disproportionately affect people living with frailty [5], prevalent amongst people living in care homes [6]. Given the potential burdens, identifying and reducing ‘inappropriate’ emergency healthcare use appears to be a sensible objective. However, in this commentary, we problematise the notion of ‘inappropriate’ emergency healthcare use and suggest that emergency healthcare use data alone do not provide a meaningful measure of the care quality provided by care homes.

### Diverse definitions of ‘inappropriate’ care use leads to varied estimates of prevalence

Despite attempts to define and differentiate between associated terms such as ‘avoidable’, ‘preventable’ and ‘unnecessary’, there remains a lack of professional and academic consensus about what constitutes ‘inappropriate’ emergency healthcare use [7–9]. Differing definitions have led to variation in estimates, from 7%–40% [3, 10]. These estimates relied on clinical outcomes—such as mortality within a defined period or clinical diagnoses for which care was sought—rather than patient- or carer-reported measures of experience. Estimates can be affected if in-hospital mortality is used to indicate inappropriateness. This fails to recognise the uncertainty of predicting deterioration trajectories and death in this population, assumes there was no value in hospital assessment and assumes that if someone is close to death, palliative treatment in the community is available, timely and preferred. Variation in estimates of appropriateness may be influenced by *who* is making a judgement [8]. Existing discourse is led by hospital-centric views, with less attention given to the views of people living and working in care homes.

### The pitfalls of using ambulatory care sensitive conditions as a proxy-measure of inappropriateness

Due to the absence of an agreed definition, attendances related to Ambulatory Care Sensitive Conditions (ACSCs) are often used as a proxy-measure of ‘inappropriateness’. ACSCs include conditions which could be: managed in the community; prevented with early intervention; and prevented through vaccination. However, measuring ACSC-related emergency healthcare use relies on the diagnoses care home residents receive *post hoc,* at the time of hospital discharge, after investigations not available in the care home have been undertaken. It is widely acknowledged that care home residents may present with atypical symptoms and may not display overt signs of being unwell. That it is later decided that the cause of symptoms did not require acute healthcare does not necessarily mean that the process of seeking help was ‘inappropriate’. The use of ACSCs as a proxy-measure assumes that, for some conditions, adequate management in the community can prevent a hospital admission. However, this may not hold true when caring for care home residents, who may have complex needs and variable access to healthcare support, particularly out-of-hours. Therefore, whilst ACSC-related emergency healthcare data can be used as a proxy-measure to assess the effectiveness of healthcare systems [11] it is unlikely to be a good measure of quality of care offered in a care home.

### Emergency healthcare use is influenced by the wider care context

Current conceptualisations of ‘inappropriate’ emergency healthcare use from care homes are based largely on quantitative data, which do not capture crucial information about the wider social context in which help was sought. This is significant because rates of emergency healthcare use can be influenced by: ‘individual factors’ related to resident characteristics; ‘organisational factors’ related to care home characteristics; and ‘structural factors’ related to healthcare system characteristics [12]. There have been repeated calls to strengthen multidisciplinary healthcare support for care home residents, to prevent staff from feeling obliged to seek emergency healthcare in instances where they could provide care on-site. This includes the NHS England Enhanced Health in Care Homes model within primary care networks [13]. Digital technologies can facilitate inter-professional communication and triage of non-urgent needs [14]. Where such additional response mechanisms are lacking, transferring a resident to hospital, even for a condition that could have been managed in the community, can be entirely appropriate. Alternative models of care, providing dedicated professional advice and support for care homes, including access to hospital at home services, can provide equitable alternatives. However, access to these services remains varied across the UK and, where they exist, not all provide 7-day services, with few resourced to respond out-of-hours.

### Professionals seeking emergency care make complex decisions

Research has uncovered the broad range of factors that staff consider when deciding whether to seek emergency healthcare [15]. This can include resident-centric factors—including the potential benefits and burdens of (not) receiving emergency healthcare, as well as non-resident-centric factors—including potential personal or professional consequences that may occur. Staff weigh-up multiple risks, often in the presence of multiple uncertainties, including to: residents; themselves (as decision-makers); their social relationships; the care home and wider system [9, 16]. Given the recognised complexity involved in staff decision-making, it is perhaps unsurprising that quality improvement initiatives focussed solely on ‘upskilling’ care home staff have shown limited impacts [4].

### The language of ‘inappropriate’ emergency healthcare use can stigmatise home residents and people who seek emergency care

The terminology used is value-laden, framing residents as ‘problematic’ for healthcare systems. Although rarely stated explicitly, this framing may reflect broader negative stereotypes that residents are older, sicker and closer to the end-of-life than the general population and therefore resources could be better directed towards others. This encourages therapeutic nihilism amongst professionals and neither reflects the diversity amongst the population living in care homes nor is it equitable. Care home residents should have the same access to emergency healthcare as people who live elsewhere in the community [13] yet extensive rhetoric around ‘inappropriate’ emergency healthcare use leads to residents being stigmatised as being ‘out of place’ in hospital settings [17]. Such language also stigmatises care home staff, by undermining the aforementioned complexity involved in their decision-making, which reinforces negative public perceptions [9]. Choosing to measure ‘inappropriate’ emergency healthcare use and using this as a marker of care home quality, must be done mindful of these negative consequences.

### Using measures to enhance understanding

Numerical data quantifying the significant healthcare needs of people living in care homes are unquestionably important to our common goal of enabling equitable access to care. We must critically assess current definitions and measures of ‘improvement’. At present, an overall reduction in emergency care attendances is often prioritised and measured in isolation from the model of external support accessible to care homes or outcomes for individuals. Outcome measures should reflect what matters to a range of stakeholders—most notably residents, but also their family carers, care home staff and healthcare professionals supporting them. Use of quantitative measures could be enhanced by the development and prioritisation of person-centred outcome and experience measures that can be routinely collected and used across health and care systems [18]. By moving away from focusing solely on the numbers of residents using emergency healthcare, through incorporating stories and experiences, we can refocus our efforts to re-design services which better support the complex needs of this population.

## Conclusion

It is crucial that we work towards ensuring residents only receive emergency healthcare when doing so would be beneficial and/or when it aligns with their wishes. We have problematised the notion ‘inappropriate’ emergency healthcare use, arguing that it is not a meaningful measure of the quality of care provided by a care home. The lack of an agreed definition leads to wide variation in estimates. Proxy-measures, such as ACSCs-related healthcare have limitations. Furthermore, the binary classification—between emergency care use that is either ‘appropriate’ or ‘inappropriate’—is over-simplistic and unable to reflect the complexity involved in decision-making and the impact of the wider healthcare system. It overlooks individual wishes and preferences, despite person-centredness being valued. Resource use data, in isolation, has the potential to further stigmatise care homes, staff and residents, who are entitled to emergency healthcare support.

Supporting residents to access healthcare support whilst in the care home, thus avoiding the harms of emergency healthcare settings, requires a tailored response. There appears to be promise in initiatives which strengthen healthcare support for care homes and those which encourage integrated working [4, 13]. Therefore, we must develop services that are resources to respond to meet residents’ individual needs, with inter-professional advice always available. Research and evaluation are needed, involving all stakeholders’ perspectives, to identify the necessary conditions for services to be implemented successfully.
